# Primitive Neuroectodermal Tumor Presenting With a Large Subcapsular Hematoma in Pregnancy: A Case Report and Current Literature Review

**DOI:** 10.7759/cureus.50359

**Published:** 2023-12-11

**Authors:** Patrick Curtin, Kyler W Perry, Gabrielle Yankelevich, Robert L Grubb

**Affiliations:** 1 Department of Urology, Medical University of South Carolina, Charleston, USA; 2 Department of General Surgery, Medical University of South Carolina, Charleston, USA

**Keywords:** ewing's sarcoma family of tumors (esft), ews-fli-1, cd-99, pregnancy, primitive neuroectodermal tumor (pnet)

## Abstract

Primitive neuroectodermal tumors (PNET) are rare, small round cell tumors that are difficult to diagnose. It is important to identify PNET early, utilizing immunohistochemistry and genetic markers, as it is often an aggressive cancer. PNET is most commonly described in men between the ages of 20 and 40, with very few case reports highlighting the development in pregnant patients. We present a case of localized renal PNET in a pregnant patient and highlight the diagnostic work-up and treatment as well as the relationship between pregnancy and the potential development of aggressive tumors.

## Introduction

Primitive neuroectodermal tumors (PNET) are classified as small round cell tumors and are difficult to differentiate from Ewing's sarcoma (EWS), Wilms' tumor (WT), neuroblastoma, and several other tumors with similar histological appearance [1]. Localized renal PNET is rare but should be included in the differential if a young adult presents with the triad of hematuria, pain, and palpable mass [2]. PNET has high metastatic potential, and the prognosis is poor. It is important to identify PNET and differentiate it from other small round cell tumors using immunohistochemistry markers such as CD-99 and genetic markers such as the* EWS-FLI-1* gene so that appropriate and aggressive treatment can decrease morbidity and mortality [2]. In this case report, we document the rare clinical presentation of localized renal PNET in a pregnant patient and discuss the appropriate diagnostic work-up and treatment.

## Case presentation

**Figure 1:**
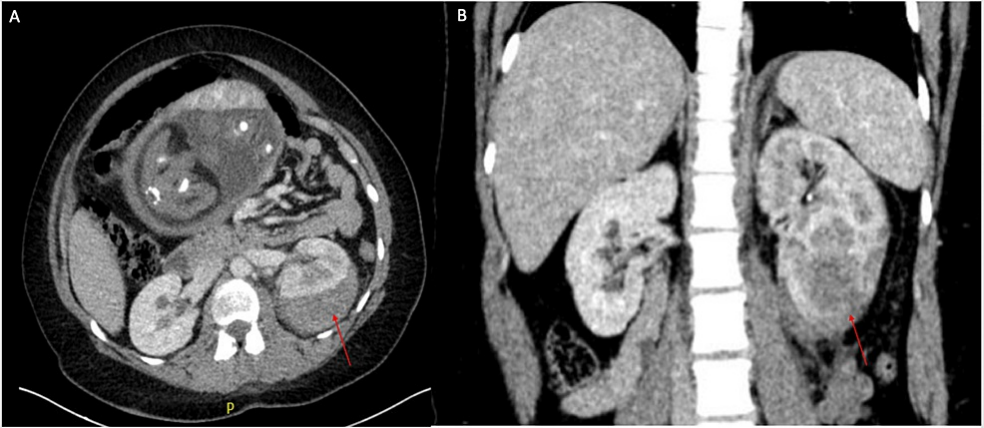
Low-dose CT showing axial (A) and coronal (B) views of the large subcapsular hematoma (red arrow)

A 25-year-old G3P1A1 with a known history of Addison's disease, bilateral nephrolithiasis, and gestational diabetes presented to the emergency department at 36 weeks' gestation with the acute onset of left-sided flank pain. A CT abdomen and pelvis was obtained due to concern for obstructing stone, which demonstrated a large left subcapsular hematoma with a delayed nephrogram (Figure 1 A,B). The suspected diagnosis at that time was hemorrhagic cyst rupture. She was admitted to the hospital for symptomatic management. Her pain was unable to be managed conservatively, so the decision was made to induce labor to facilitate further management of the renal hematoma. She had an uncomplicated vaginal delivery, and the baby was without abnormalities. Afterwards, her pain was adequately controlled, and her labs and vitals were stable, so she was discharged from the hospital with plan for outpatient interval imaging. 

**Figure 2:**
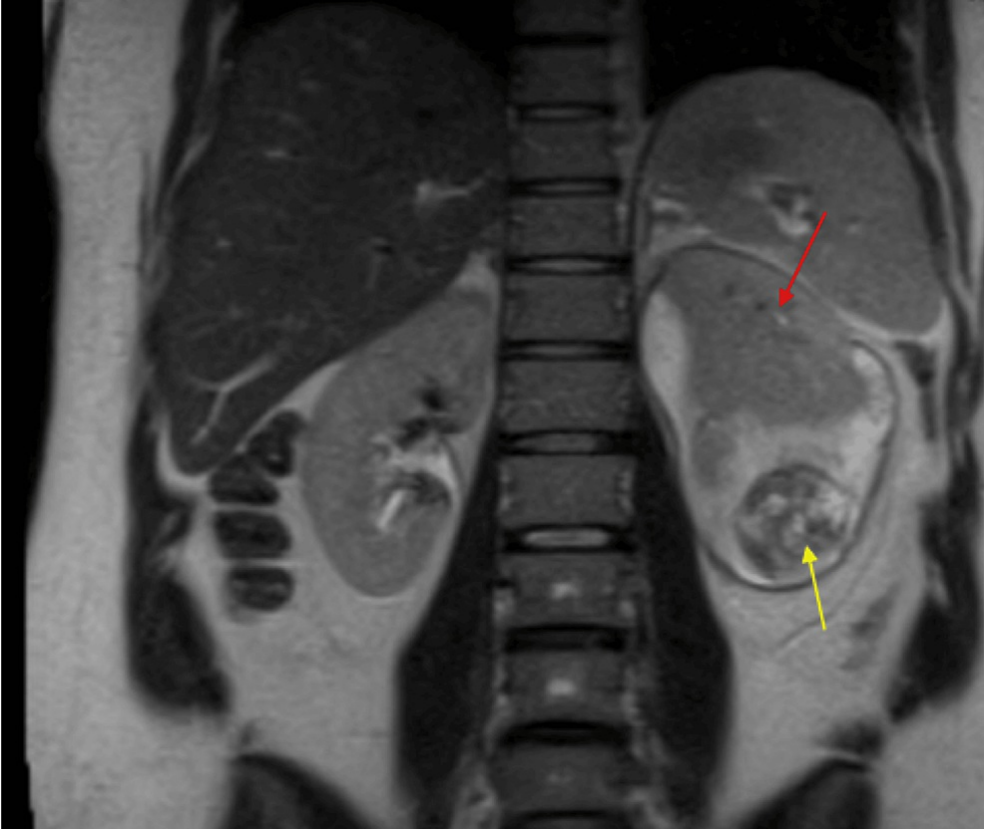
Coronal T2-weighted MRI abdomen showing left-sided inferior pole renal mass (yellow arrow) and subcapsular hematoma (red arrow)

Eleven days postpartum, the patient re-presented to the emergency room with uncontrolled flank pain, dysuria, hematuria, and intermittent fevers. CT showed a persistent subcapsular fluid collection in the left kidney. MRI abdomen and pelvis with contrast confirmed a 3.9 x 2.5 cm enhancing lesion in the inferior pole of the left kidney with associated subcapsular hematoma measuring 8.5 cm in the greatest dimension and associated compression of the kidney parenchyma (Figure 2). After presentation in tumor board, the decision was made to pursue a CT-guided biopsy of the left renal mass. Pathology revealed PNET with positive staining for FLI-1 and CD99. A metastatic work-up including contrasted CT of the chest was negative for metastatic disease. After a discussion of the risks and benefits of neoadjuvant chemotherapy with a cisplatin-based regimen, the patient elected for immediate surgical resection with plan for adjuvant chemotherapy. A left open radical nephrectomy and adrenalectomy was performed. Histopathology and immunohistochemistry of the mass confirmed a 7 cm mass with negative margins and staining consistent with high-grade PNET. She tolerated the procedure well and was discharged from the hospital on postoperative day 4.

Due to the adverse pathologic features, her case was discussed at the multidisciplinary tumor board, and she was recommended adjuvant chemotherapy with dose-intense vincristine, doxorubicin, and cyclophosphamide (VDC) and ifosfamide and etoposide (IE) every two weeks for a total of five cycles. At most recent follow-up, 24 months postoperatively, she remains disease-free.

## Discussion

Primary renal PNET is an extremely rare clinical entity that must be distinguished from more common renal cell carcinomas as well as from histologically similar carcinomas like EWS, WT, and neuroblastoma [1]. While patients with PNET may present with distant metastases at initial presentation, it is most commonly seen in young men between the ages of 20 and 40 who present with flank pain and hematuria [3-5]. The most common sites of metastases include the lung, liver, and abdominal lymph nodes [3]. The aggressive nature of this disease and the proclivity for advanced disease at presentation result in a 60-70% five-year survival for patients with non-metastatic disease and 20-30% five-year survival for patients with metastatic disease [5-7]. 

Differentiating PNET from its far more common renal cancer counterparts is extremely important due to high likelihood of presenting with or progressing to advanced disease. Initial work-up should include history and physical as well as appropriate cross-sectional imaging. As large renal masses are otherwise uncommon in this age group population, renal biopsy should be completed with appropriate pathologic evaluation. In this case, staining should include CD-99, a nonspecific marker for members of Ewing's sarcoma family of tumors (ESFT), as well as FL-1, part of the more specific EWS-FL1 fusion oncogene present in up to 90% of PNET/EWS tumors [8].

Although no standard of care exists for the treatment of PNET due to its rarity, most clinicians advocate for a combination of neoadjuvant chemotherapy and local surgical control with adjuvant chemotherapy [9-10]. In a case series for PNET of the thorax, neoadjuvant chemotherapy led to a significant increase in the ability to complete resection of the tumor and an increase in overall survival of greater than 30% [10]. Much of the clinical dogma surrounding the treatment of PNET is derived from regimens developed for other members of the ESFT such as EWS of the bone, with current recommendations advocating for neoadjuvant chemotherapy with a combination of VDC/IE before surgical resection or radiotherapy [11]. 

Similar to the 2019 case report published by Findlay et al., our patient elected to forgo neoadjuvant chemotherapy and immediately undergo surgical resection secondary to poor pain control [3]. Of note, this same study advocates for the completion of renal biopsy for prognostic purposes, which our patient underwent. Despite electing immediate surgical resection, the multidisciplinary team felt that biopsy should be offered as standard of care as it significantly increases the utility of chemotherapeutic agents and evidence should be included in any care discussion.

PNET/EWS in pregnant patients is extremely rare with few case reports in publication discovered during our literature review [12]. Sites of origin have included the brain, pancreas, ovary, and cervix, with a select number of recent case reports with primary renal tumors [12]. Pregnancy represents a unique state in terms of immune function as the mother's body must seek to balance protection from foreign antigens and infections with tolerance of the foreign fetal antigens. The exact relationship between the development of EWS/PNET and pregnancy has yet to be determined. 

## Conclusions

Renal PNET is a rare diagnosis, and there are even fewer examples of this pathology occurring in pregnancy. The work-up of these cases differ from classic renal cell carcinomas, as biopsies should be performed and neoadjuvant or adjuvant therapy should be utilized in conjunction with surgical resection. The final diagnosis rests on pathologic and immunohistochemical evaluation. Ultimately, rare case presentations underscore the importance of maintaining a broad differential diagnosis. 
